# Loss over 5% of chromosome 1p is a clinically relevant and applicable cut-off for increased risk of recurrence in meningioma

**DOI:** 10.1007/s00401-024-02777-z

**Published:** 2024-08-08

**Authors:** Sybren L. N. Maas, Thomas Hielscher, Philipp Sievers, Volker Hovestadt, Abigail K. Suwala, Till Acker, Michael Weller, Matthias Preusser, Christel Herold-Mende, Wolfgang Wick, Andreas von Deimling, Natalie Berghaus, Felix Sahm

**Affiliations:** 1 Department of Pathology, Leiden University Medical Center, Leiden, Netherlands; 2 Department of Pathology, Erasmus MC Cancer Institute, University Medical Center Rotterdam, Rotterdam, The Netherlands; 3 Department of Biostatistics, German Cancer Research Center (DKFZ), Heidelberg, Germany; 4 Department of Neuropathology, German Consortium for Translational Cancer Research (DKTK), German Cancer Research Center (DKFZ), University Hospital Heidelberg and CCU Neuropathology, Heidelberg, Germany; 5 Department of Pediatric Oncology, Dana-Farber Cancer Institute, Boston, MA USA; 6 Division of Hematology/Oncology, Boston Children’s Hospital, Boston, MA USA; 7 Broad Institute of MIT and Harvard, Cambridge, MA USA; 8 Institute of Neuropathology, Justus-Liebig-University, Gießen, Germany; 9 Department of Neurology, Clinical Neuroscience Center, University Hospital and University of Zurich, Zurich, Switzerland; 10 Division of Oncology, Department of Medicine I, Medical University of Vienna, Vienna, Austria; 11 Division of Experimental Neurosurgery, Department of Neurosurgery, University Hospital Heidelberg, Heidelberg, Germany; 12 Clinical Cooperation Unit Neurooncology, German Consortium for Translational Cancer Research (DKTK), German Cancer Research Center (DKFZ), Heidelberg, Germany; 13 Department of Neurology and Neurooncology Program, National Center for Tumor Diseases, Heidelberg University Hospital, Heidelberg, Germany

Copy-number variations (CNVs) in meningioma have been studied for decades: > 50% of meningioma show 22q loss, the chromosomal arm that harbors the *NF2*-gene [2, 7, 10, 11]. Other frequent CNVs include losses in 1p, 6p/q, 10q, 14q and 18p/q. Investigations into the temporal order of CNVs, identified 1p loss as the first CNV after 22q loss [7, 8]. Complimenting earlier research on 1p loss as a meningioma risk factor [1, 4, 5], recent studies including all CNVs, revealed that 1p loss was an independent risk factor when correcting for age, CNS WHO grade, tumor location and other CNVs [6, 7]. Therefore concurrent 1p and 22q loss is now discussed as a molecular criterion to identify cases at higher risk despite benign morphology. However, different cut-off levels have been proposed to determine the (segmental) loss of 1p as a relevant CNV (e.g., 5 versus 30%) [2, 7]. Pragmatically, we have previously termed the loss of 1p as “complete” when > 95% of the chromosomal arm was deleted, while losses between > 5 and 95% were classified as “segmental”[3, 7]. The percentages are obtained from epigenetic analyses processed by the (expanded) conumee package for R (https://github.com/dstichel/conumee/). Recurrence risk for complete and segmental losses were similar and therefore > 5% loss was identified as predictive for recurrence [3]. So far, it is unknown if other cut-offs are more suitable and more accurately predict meningioma outcome. To this end, we here query data from 2257 meningioma in highest granularity, including three clinical cohorts [3, 7] to explore the potential for alternative thresholds.

**Fig. 1:**
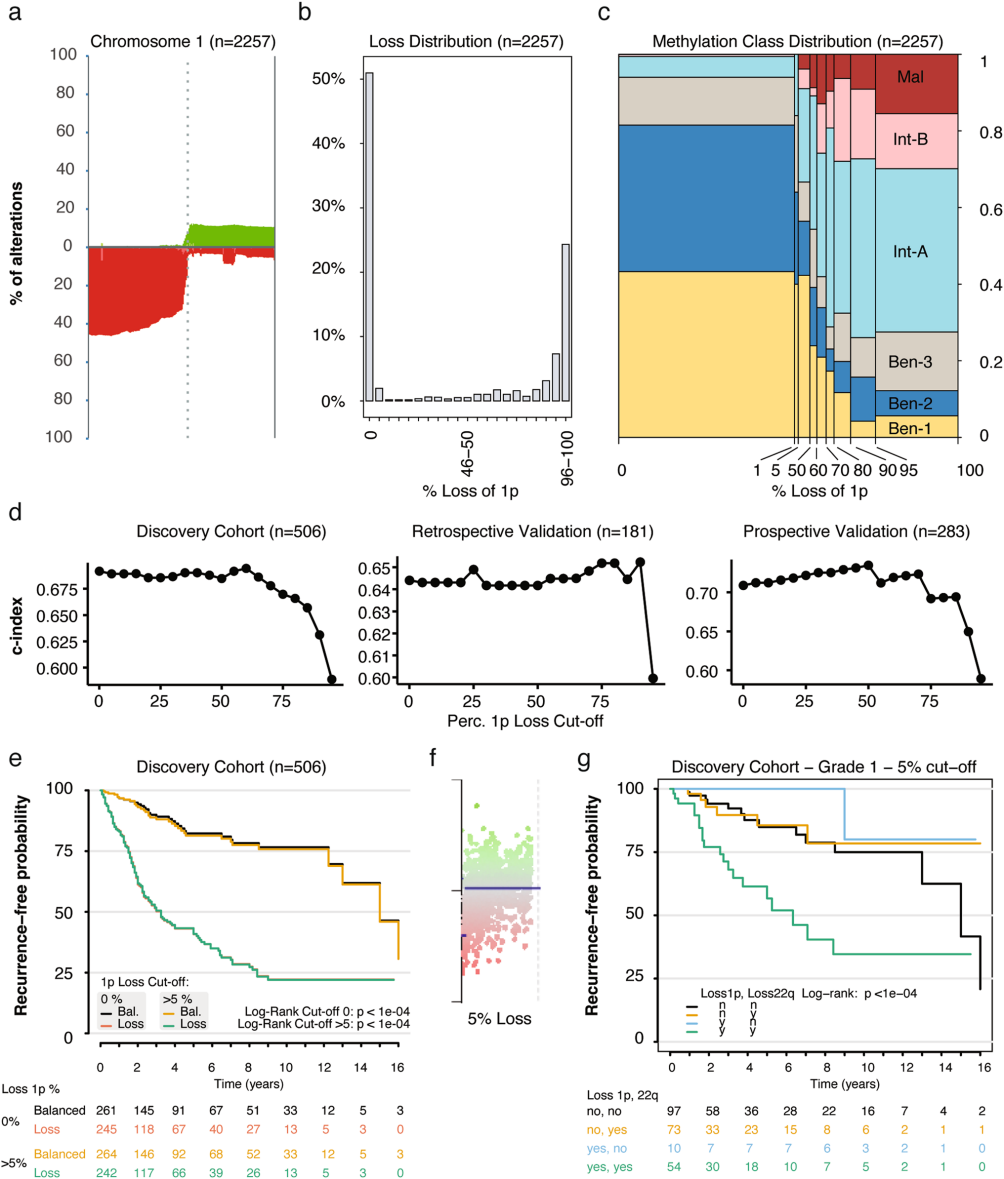
Chromosome 1 losses and gains in 1% bins for 2257 meningioma **a**. 1p loss distribution over 5% bin intervals **b**. Epigenetic methylation class (MC) distribution over the CNV-bin intervals **c**. Prognostic c-index at different cut-offs in three independent meningioma cohorts **d**. Kaplan–Meier at a 0% or 5% cut-off differentiates low and high-risk cases **e**. Example case indicating that a cut-off below 6% may lead to false positive loss detection **f**. CNS WHO grade 1 cases with concurrent 1p and 22q loss (> 5% loss) have a significant reduced recurrence − free probability **g**

First, we investigated the extend of chromosome 1 loss in 2257 meningioma, spanning different CNS WHO grades and epigenetic methylation classes (described below), for which data were available in the Heidelberg University Neuropathology database. Here, 1p loss slightly increases toward the telomere suggesting that these losses may be missed with more centrally located FISH-probes. (Fig. 1b). By binning the loss percentage into 5% segments, it is identified that the majority of cases have either 0% or 96–100% loss of chromosome 1p (Fig. 1b). Meningioma consist of six epigenetic methylation classes (MCs) [9]. Three classes (MC Ben-1/Ben-2/Ben-3) are associated with relatively low risk for progression, two classes (MC Int-A/-B) with intermediate risk and one class (MC Mal) with high risk for progression. To understand the relationship between 1p loss and MC, we plotted the MC distribution over the 1p loss percentage (Fig. 1c). Here it was observed that 94% of cases with 0% loss are of the Ben-1/Ben-2/Ben-3 MCs, suggesting a strong association of minimal chromosomal disruption with lower-risk classifications. Conversely, the percentage of the MCs associated with higher risk (i.e., MC Int-A, Int-B and Mal) gradually increases to 72% of cases with over 95% loss of chromosome 1p. These results illustrate the relation between methylation-based risk stratification and extent of chromosomal 1p loss in meningioma.

To investigate how the cut-off influences outcome discrimination as determined by the so called “c-index”, we compared the c-index by splitting cases at different cut-offs in three independent meningioma cohorts (Fig. 1d). This analysis suggested that lower cut-offs, which consider any detectable 1p loss as significant, might be most effective in differentiating patient outcomes. To further test this, a Kaplan–Meier plot of the discovery cohort was generated with a cut-off at 0%. This means that all cases with a loss over 0% are considered a loss. Here, a significant difference was observed with a difference of 11.8 years in median recurrence free time (Fig. 1e). Similar differences were observed for the retrospective and prospective validation cohorts (data not shown). These data suggest that any loss over 0%, can be used as a marker for increased risk in meningioma. The biologic reason for this remains to be elucidated, however, we hypothesize that even small losses are indicative for chromosomal instability and thus increased growth. In clinical practice, the results from a methylation array are not assessed using quantitative bioinformatic pipelines, but the loss of 1p is determined by visual inspection of the CNV-plot provided by the online brain tumor classifier or similar tools. Therefore, we inspected multiple CNV-plots of cases identified with losses between 0 and 15% from the three cohorts. This yielded multiple cases where the loss percentage was either based on noise or very focal losses almost impossible to identify by eye (e.g. Figure 1f). Therefore, since losses over 5% were identifiable by eye, we propose to determine a clinically relevant loss cut-off for chromosome 1p at 5% (i.e., counting cases with a loss percentage of 6% or higher). This adjustment affected a small number of cases as 1–5% 1p loss was only detected in 44 out of the 2257 (1.95%) cases included. Additionally, no significant difference in the Kaplan–Meier plot was observed for the different cut-offs of 0 and 5% (Fig. 1e). To confirm the clinical relevance of the 5% cut-off, we investigated cases in the discovery cohort with a histologic CNS WHO grade 1 meningioma. Here we split the cases based on the presence or absence of chromosomal 1p and 22q losses as determined by the 5% cut-off. This investigation revealed that CNS WHO grade 1 meningioma with a concurrent 1p and 22q loss (6% or more of the chromosomal arm) have significant higher risk for progression compared to cases with no or single 1p/22q losses (Fig. 1g).

In conclusion, this study identifies that 1p loss of 6% or higher, as identified by epigenetic profiling, as an increased risk in meningioma is clinically relevant and applicable. Although technically all losses over 0% can be used to predict risk, technical noise of the analysis may result in overcalling meningioma ‘at risk’. Specific cut-offs for different modes of CNVs investigation will have to be established separately.
